# Serum vascular endothelial growth factor-D as a diagnostic and therapeutic biomarker for lymphangioleiomyomatosis

**DOI:** 10.1371/journal.pone.0212776

**Published:** 2019-02-28

**Authors:** Masaki Hirose, Akiko Matsumuro, Toru Arai, Chikatoshi Sugimoto, Masanori Akira, Masanori Kitaichi, Lisa R. Young, Francis X. McCormack, Yoshikazu Inoue

**Affiliations:** 1 Clinical Research Center, National Hospital Organization Kinki-Chuo Chest Medical Center, Sakai, Osaka, Japan; 2 Department of Radiology, National Hospital Organization Kinki-Chuo Chest Medical Center, Sakai, Osaka, Japan; 3 Department of Pathology, National Hospital Organization Minami Wakayama Medical Center, Tanabe, Wakayama, Japan; 4 Vanderbilt University Medical Center, Nashville, Tennessee, United States of America; 5 University of Cincinnati College of Medicine, Cincinnati, Ohio, United States of America; Children's Hospital of Los Angeles, UNITED STATES

## Abstract

**Background:**

In lymphangioleiomyomatosis (LAM), tuberous sclerosis gene mutations activate the mechanistic target of the rapamycin pathway, resulting in vascular endothelial growth factor-D (VEGF-D) overproduction. While the utility of serum VEGF-D testing for the diagnosis of LAM is outlined in ATS/JRS LAM Guidelines, the assay has not been fully validated for Asian populations. Our aims were to validate serum VEGF-D testing in Japan, by directly comparing measurements in Japan and the U.S., determining the diagnostic cut-off for serum VEGF-D levels among the Japanese women with typical thin walled cystic change on CT, and determining the performance of VEGF-D as a prognostic biomarker.

**Subjects and methods:**

We determined serum VEGF-D levels from 108 LAM patients, 14 disease controls, and 51 healthy volunteers from the Japanese population. Measurements of 61 LAM patients were compared to those from the principal VEGF-D laboratory in the U.S at Cincinnati Children’s Hospital Medical Center. We correlated baseline serum VEGF-D levels with baseline and longitudinal clinical data to determine how pregnancy, sirolimus or gonadotrophin-releasing hormone (GnRH) agonists influence serum VEGF-D levels.

**Results:**

Serum VEGF-D measurements in Japan and the U.S. were very similar. Baseline serum VEGF-D levels effectively distinguished LAM from other diseases and healthy volunteers at a cut-off level of 645 pg/ml and were diagnostically specific at 800 pg/ml, consistent with the recommendations of the ATS/JRS LAM Guidelines. Baseline serum VEGF-D correlated negatively with the DLco baseline % predicted and with the annual decrease in DLco % predicted. There was no significant association between baseline serum VEGF-D level and the outcomes of death or transplant. Serum VEGF-D levels markedly decreased during treatment with sirolimus, but not with GnRH analogues. Serum VEGF-D levels of most LAM patients did not increase over time, and neither pregnancy nor menopause significantly modulated serum VEGF-D levels.

**Conclusions:**

Serum VEGF-D is a useful diagnostic and therapeutic biomarker for LAM. Satisfactory precision and international inter-laboratory agreement of the clinical assay support VEGF-D recommendations in the ATS/JRS LAM Guidelines for the Japanese population.

## Introduction

Lymphangioleiomyomatosis (LAM) is a rare and progressive cystic lung disease that primarily affects premenopausal women. It can occur sporadically (S-LAM) or in association with tuberous sclerosis complex (TSC-LAM), a heritable tumor suppressor syndrome caused by *TSC1* or *TSC2* germ line mutations. TSC-LAM typically presents with seizures, cognitive impairment, and neoplastic growths in multiple organs, including renal angiomyolipomas (AML) [1, 2]. S-LAM is also associated with *TSC1* and *TSC2* somatic mutations of LAM cells. Hamartin and tuberin, the gene products of *TSC1* and *TSC2*, respectively, form a complex that suppresses the signaling effector, mechanistic target of rapamycin (mTOR). LAM cells harboring *TSC* mutations exhibit constitutive mTOR activation and metastasize to the lung from an unknown source, resulting in tissue remodeling that leads to cystic changes [3]. LAM cells also express elevated levels of serum vascular endothelial growth factor (VEGF)-D, a growth factor this in known to promote active tumor lymphangiogenesis and spread to regional lymph nodes for other neoplasms [4].

Young et al. first described the utility of VEGF-D for diagnosing LAM in women with typical cystic change on High-Resolution Computed Tomography (HRCT) [5, 6] and the diagnostic performance of the test has since been validated in several LAM cohorts [7–10]. High serum VEGF-D levels have been associated with lymphatic manifestations [11, 12] and more rapid disease progression [13] in LAM patients. The findings that LAM cells use lymphangiogenesis as a strategy for metastatic spread and tissue remodeling suggest that VEGF-D may directly contribute to the neoplastic potential of LAM [14].

The Multicenter International (U.S., Japan, Canada) LAM Efficacy and Safety of Sirolimus (MILES) trial demonstrated that sirolimus, an mTOR inhibitor, stabilized lung function and improved functional performance and quality of life in patients with LAM [8]. Baselined VEGF-D levels correlated with baseline markers for disease severity and decreased by more than 50% with sirolimus treatment. In addition, higher baseline serum VEGF-D levels were associated the rate of forced expiratory volume in one second (FEV_1_) decline, such that higher levels correlated with more rapid disease progression in the placebo group and with better treatment response in the sirolimus group. Collectively, these findings support the use of serum VEGF-D levels as a diagnostic and predictive biomarker [13]. Recently, the American Thoracic Society (ATS) and Japanese Respiratory Society (JRS) published joint guidelines recommending that VEGF-D testing be used for diagnosing LAM before considering surgical lung biopsy [15, 16]. However, this clinical assay has not been directly validated for the Japanese population.

The aims of the present study were to validate the precision and international inter-laboratory agreement of VEGF-D measurements and to clarify the diagnostic, prognostic and predictive value of baseline and longitudinal serum VEGF-D levels under several conditions, including pregnancy, menopause, and treatment with sirolimus or GnRH agonists, in our Asian cohort.

## Materials and methods

### Subjects

Serum VEGF-D levels were measured in 108 female LAM patients (S-LAM, 92; TSC-LAM, 16), 14 females with other cystic or lymphatic lung diseases, and 51 healthy female volunteers between October 2007 and November 2016. The cohort with other lung diseases included patients with Langerhans cell histiocytosis (n = 4), chronic obstructive pulmonary disease (n = 4), plasma cell variant of Castleman's disease (n = 2), lymphoproliferative disease (n = 1), lymphangiomatosis (n = 1), spontaneous pneumothorax (n = 1) and emphysema and bullae (n = 1). Among all LAM patients, 61 received drug treatments for LAM during the study period, including 35 who received sirolimus and 26 who received hormone therapy (Leuprorelin acetate, 14; Buserelin acetate, 12). Four patients became pregnant during the study period.

Patients were diagnosed with LAM or other lung diseases at the National Hospital Organization Kinki-Chuo Chest Medical Center between 1991 and 2013. The diagnosis of LAM was established and confirmed based on diagnostic criteria outlined in the ATS/JRS LAM Clinical Practice Guidelines [8]. Sixty-nine LAM patients were diagnosed by surgical lung biopsy, 18 LAM patients by transbronchial lung biopsy, and 21 LAM patients without chylous ascites, chylous pleural effusion, or AML were diagnosed by multi-disciplinary discussion by radiologist and/or pathologist and/or clinician using serum VEGF-D level as a diagnostic biomarker.

We obtained written informed consent from all subjects and this study was approved by the Institutional Review Board (IRB) of the National Hospital Organization Kinki-Chuo Chest Medical Center (IRB #365). To compare serum VEGF-D levels measured in both the Kinki-Chuo and U.S. labs, we used samples and data from MILES trial subjects at our institution (IRB # 192).

### Measurement of serum VEGF-D levels

Serum VEGF-D levels were measured using a commercially available ELISA kit (R&D Systems Inc., Minneapolis, MN, USA) according to the manufacturer’s protocol [5, 17]. Assay precision was determined using intra- and inter-assay variability testing. For the intra-assay variability assessment, we analyzed 10 replicates of two samples on the same plate. Inter-assay variability was assessed by running two samples on 10 different plates. To determine international inter-institutional assay variability, serum VEGF-D levels were measured using stored serum samples from our Osaka MILES patients, both in our laboratory and the TTDSL. The TTDSL remains the only College of American Pathologists/Clinical Laboratory Improvement Amendments (CAP/CLIA) approved laboratory performing VEGF-D testing in the U.S.

### Pulmonary function testing

Pulmonary function tests were performed by certified pulmonary function technicians, using a CHESTAC-8800 (Chest M.I., Inc., Tokyo, Japan) according to ATS/European Respiratory Society (ERS) approved protocols [8, 18, 19]. The data collected included FEV_1_, forced vital capacity (FVC), single-breath diffusing capacity of monoxide (DL_co_), total lung capacity (TLC), and residual volume (RV). Each pulmonary function measurement was expressed as a percent predicted value, according to the reference equations endorsed by the JRS [20].

### Statistical analysis

We performed statistical analyses using JMP software, version 10.0.2 (SAS Institute, Cary, NC, USA. Much of the data in this study did not show a normal distribution. We expressed numeric variables as the median and interquartile range (IQR), and differences in baseline VEGF-D levels between each item were determined using the Wilcoxon rank-sum test. To obtain cut-off serum VEGF-D levels for LAM diagnosis, we developed a receiver operating characteristic (ROC) curve by plotting the sensitivity of the assays against false positivity (1-specificity). The difference between the median baseline serum VEGF-D levels and those at 6 and 12 months was assessed using the Wilcoxon signed-rank test. A log rank test was used in the Kaplan-Meier analysis to assess the association between serum VEGF-D levels and time to death or lung transplant. Differences were considered significant if p < 0.05.

## Results

### Characteristics of subjects

Table 1 shows the clinical characteristics and pulmonary function test results of patients with S-LAM and TSC-LAM. There was no significant difference in the age of patients with S-LAM (median, 40 years; range, 34–37 years) and TSC-LAM (median, 36 years; range, 29–49 years) compared with the group of patients with other lung diseases (median, 35 years; range, 28–45 years) and healthy volunteers (median, 42; range, 26–49 years).

**Table 1:** The demographic and clinical characteristics of subjects.

		S-LAM		TSC-LAM	p-value
Number of women		92		16	
Age—yr (IQR)		40 (34–47)		36 (29–49)	0.77
Onset of symptoms—age (IQR)		35 (31–45)		28 (7–48)	0.14
Follow-up period—months (IQR)		52.8 (36.3–73.7)		69.5 (27.2–83.4)	0.55
Smoking					0.29
Current		3		1	
Former		21		1	
Never		68		14	
Extra pulmonary lesions—n (%)					
renal angiomyolipoma		18 (19.4)		13 (81.3)	<0.0001
extrarenal angiomyolipoma		6 (6.5)		0 (0)	0.29
lymphangioleiomyoma		28 (30.1)		0 (0)	0.01
pneumothorax		41 (44.0)		6 (37.5)	0.6
chylothorax		5 (5.4)		0 (0)	0.34
Treatment—n (%)					
GnRH analogue therapy		25 (27.2)		2 (12.5)	0.21
post menopause		37 (40.0)		6 (37.5)	0.83
mTOR inhibitor therapy		33 (35.5)		2 (12.5)	0.07
supplemental oxygen		19 (20.4)		5 (31.3)	0.35
Pulmonary function tests	n		n		
FEV_1_, % predicted	85	75.3 (55.1–103.9)	12	80.1 (53.3–96.5)	0.93
FVC, % predicted	85	99.0 (85.4–112.5)	12	95.4 (80.2–117.2)	0.88
DLco, % predicted	81	58.8 (36.4–75.5)	11	51.6 (39.5–78.3)	0.91
TLC, % predicted	81	107.9 (98.0–120.2)	10	95.7 (86.3–131.3)	0.51
RV, % predicted	77	139.1 (115.4–165.7)	10	129.0 (88.6–194.7)	0.37

Definitions of abbreviations: LAM, lymphangioleiomyomatosis; TSC, tuberous sclerosis complex; GnRH, gonadotropin releasing hormone; mTOR, mechanistic target of rapamycin; FEV_1_, forced expiratory volume in 1 second; FVC, forced expiratory volume; DLco, diffusing capacity for carbon monoxide; TLC, total lung capacity; RV, residual volume. Data are expressed as median (interquartile range, IQR). The difference between groups was analyzed using the Wilcoxon rank-sum test.

### Measurement of serum VEGF-D levels

**Fig 1:**
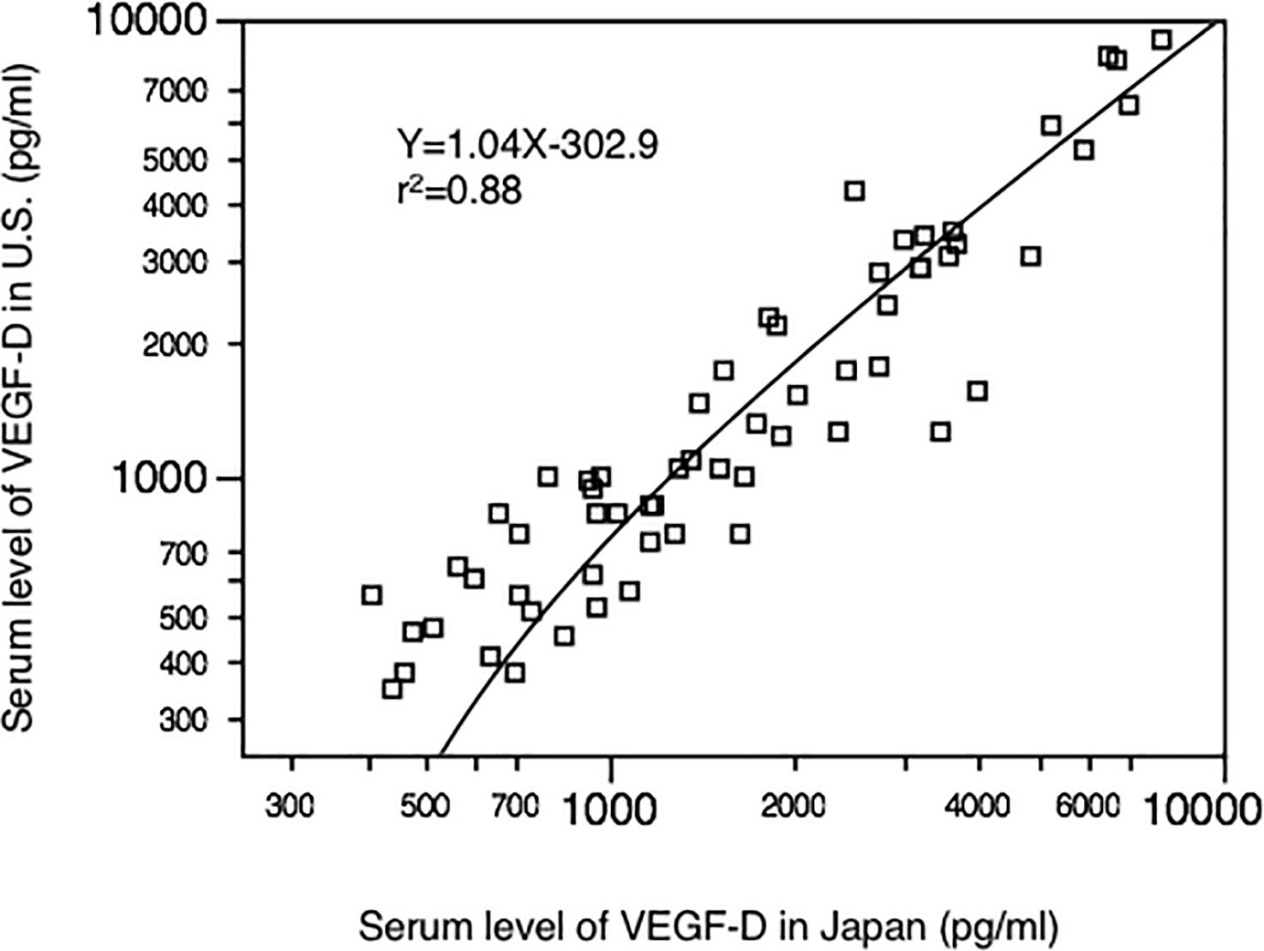
Agreement in VEGF-D measurements between U.S. and Japanese laboratories using the same ELISA kit (R&D Systems Inc.) and the same samples from Osaka MILES patients (n = 61).

The coefficients of variation for intra- and inter-assay variability were 2.33% (n = 10) and 5.38% (n = 10), respectively. Furthermore, we found that the international inter-institutional agreement of serum VEGF-D levels for samples tested in both Japan and the U.S. was excellent (r^2^ = 0.88, n = 61; Fig 1). Out of 108 LAM patients, 61 samples of 19 patients who participated in MILES trial were compared.

**Fig 2:**
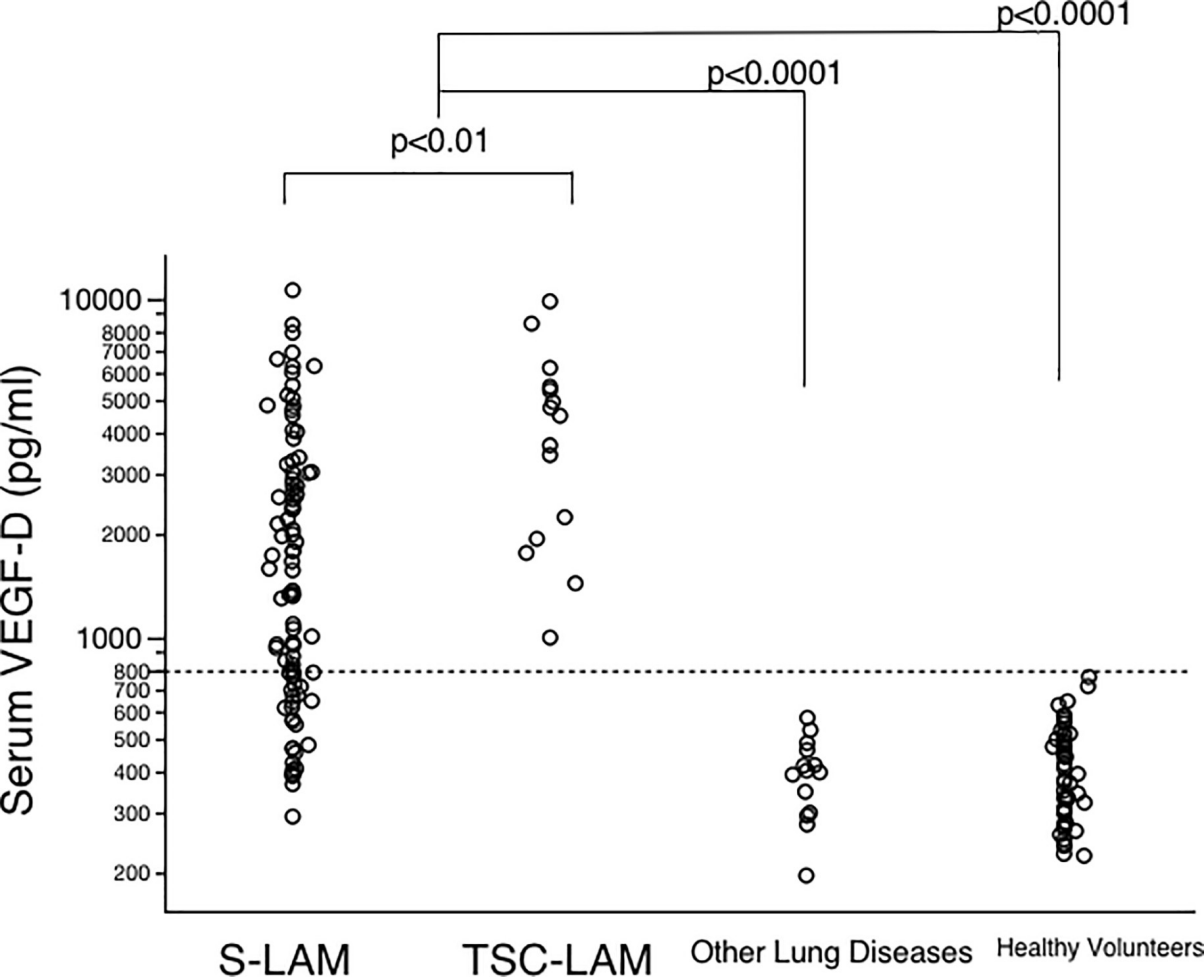
Serum VEGF-D levels in 108 LAM patients (sporadic, 92; TSC, 16) compared to the levels of 14 female patients with other chylous and cystic lung diseases and 51 healthy female volunteers. The dotted line shows the diagnostic threshold of 800 pg/ml. S-LAM, sporadic LAM; TSC-LAM, tuberous sclerosis complex-LAM. Data were analyzed using the Wilcoxon rank-sum test.

Fig 2 shows the distribution of serum VEGF-D levels among LAM patients, patients with other lung diseases, and healthy volunteers. Levels were significantly elevated in LAM patients compared with patients with other lung diseases (p < 0.0001) and healthy volunteers (p < 0.0001). The median serum VEGF-D level was 1568 pg/ml (range, 294–10,536 pg/ml) in S-LAM patients, 4485 pg/ml (range, 992–9765 pg/ml) in TSC-LAM patients, 399 pg/ml (range, 197–575 pg/ml) in patients with other lung diseases, and 392 pg/ml (range, 225–759 pg/ml) in healthy volunteers. Serum VEGF-D levels were significantly higher among TSC-LAM patients compared to those with S-LAM (p < 0.001). There was no significant difference between the median serum VEGF-D levels of healthy women and healthy mend (S1 Fig).

Lymphangioleiomyomas, enlargement of lymph nodes and leg swelling were more common in patients with elevated serum VEGF-D levels, but were not correlated with any other clinical features, including history of renal AML, extra-renal AML, chylothorax, pneumothorax, or menopause (Table 2).

**Table 2:** Association between clinical features and serum VEGF-D levels.

	Yes	VEGF-D (pg/ml)	No	VEGF-D (pg/ml)	p-value
Renal angiomyolipoma	31	1943.1	77	1789.7	0.70
Lymphangioleiomyoma	28	2800.6	80	1323.1	< 0.001 *
Chylothorax	5	2818.5	103	1783.1	0.29
Pneumothorax	47	1331.1	61	2003.7	0.32
Menopause	43	1364.7	65	2067.7	0.56
Supplemental oxygen therapy	24	2697.1	84	1750.4	0.13

Definitions of abbreviations: VEGF-D, vascular endothelial growth factor-D. Median VEGF-D

level of each group is shown. Characteristics were analyzed using the Wilcoxon rank-sum test.

### Serum VEGF-D as a diagnostic biomarker for LAM in a Japanese cohort

**Fig 3:**
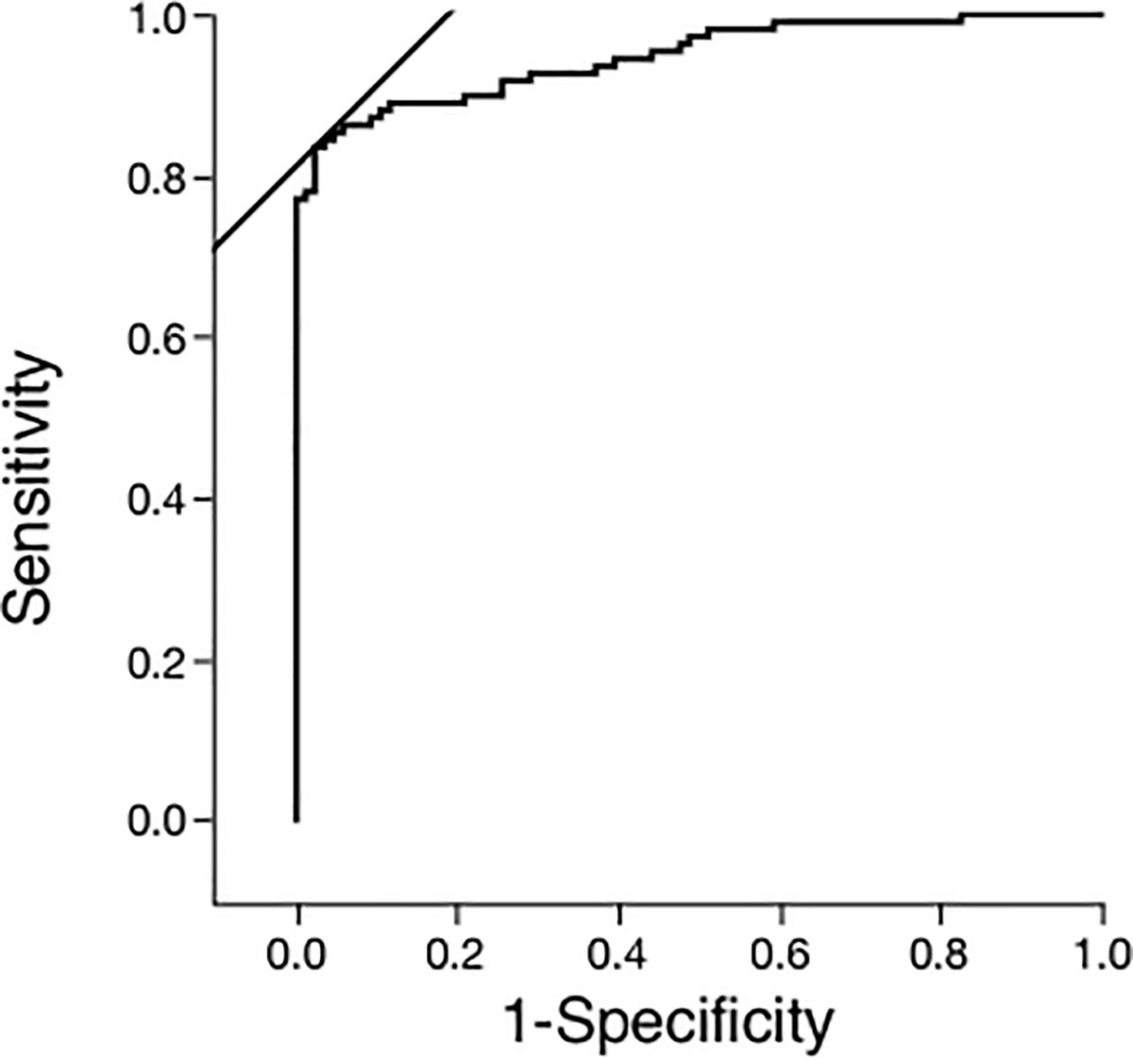
Utility of serum VEGF-D for diagnosing LAM was determined using a receiver operative characteristics (ROC) curve analysis comparing sensitivity and specificity. The area under the curve and the cut-off level were 0.94 and 645.0 pg/ml, respectively.

Serum VEGF-D showed excellent test performance as a diagnostic biomarker based on our receiver operating characteristic (ROC) curve (Fig 3). At a cut-off level of 645 pg/ml, the sensitivity for diagnosing LAM was 0.83 and the specificity was 0.97. At 700 pg/ml and 800 pg/ml, the sensitivity and the specificity were 0.80 and 0.97, and 0.72 and 1.00, respectively.

### Baseline serum VEGF-D levels and baseline and annual change of pulmonary function

Baseline serum VEGF-D levels were significantly negatively correlated with percent predicted DL_co_ (ρ = −0.29, n = 94, p = 0.007) and yearly percent decrease in DLco (Δ%DLco/year; ρ = −0.22, n = 77, p = 0.04). No other baseline or longitudinal lung function parameter correlated with baseline serum VEGF-D levels (Table 3).

**Table 3:** Correlation between baseline serum VEGF-D levels and pulmonary function tests at baseline and over time.

Baseline pulmonary function test results			
	N	ρ	p-value
%FEV_1_	97	–0.17	0.10
%FVC	97	–0.01	0.95
%DLco	94	–0.29	< 0.01 *
%TLC	91	0.05	0.61
%RV	88	–0.004	0.97
FEV_1_/FVC	82	–0.16	0.13
Rate of change in pulmonary function tests over time			
Δ%FEV_1_/year	80	–0.1	0.34
Δ%FVC/year	80	–0.16	0.15
Δ%DLco/year	77	–0.22	0.04*
Δ%TLC/year	74	–0.13	0.29
Δ%RV/year	71	–0.06	0.65
Δ%FEV_1_/FVC/year	78	–0.01	0.91

Definitions of abbreviations: FEV_1_, forced expiratory volume in 1 second; FVC, forced expiratory volume; DLco, diffusing capacity for carbon monoxide; TLC, total lung capacity; RV, residual volume.

### Baseline serum VEGF-D levels and prognosis in LAM

**Fig 4:**
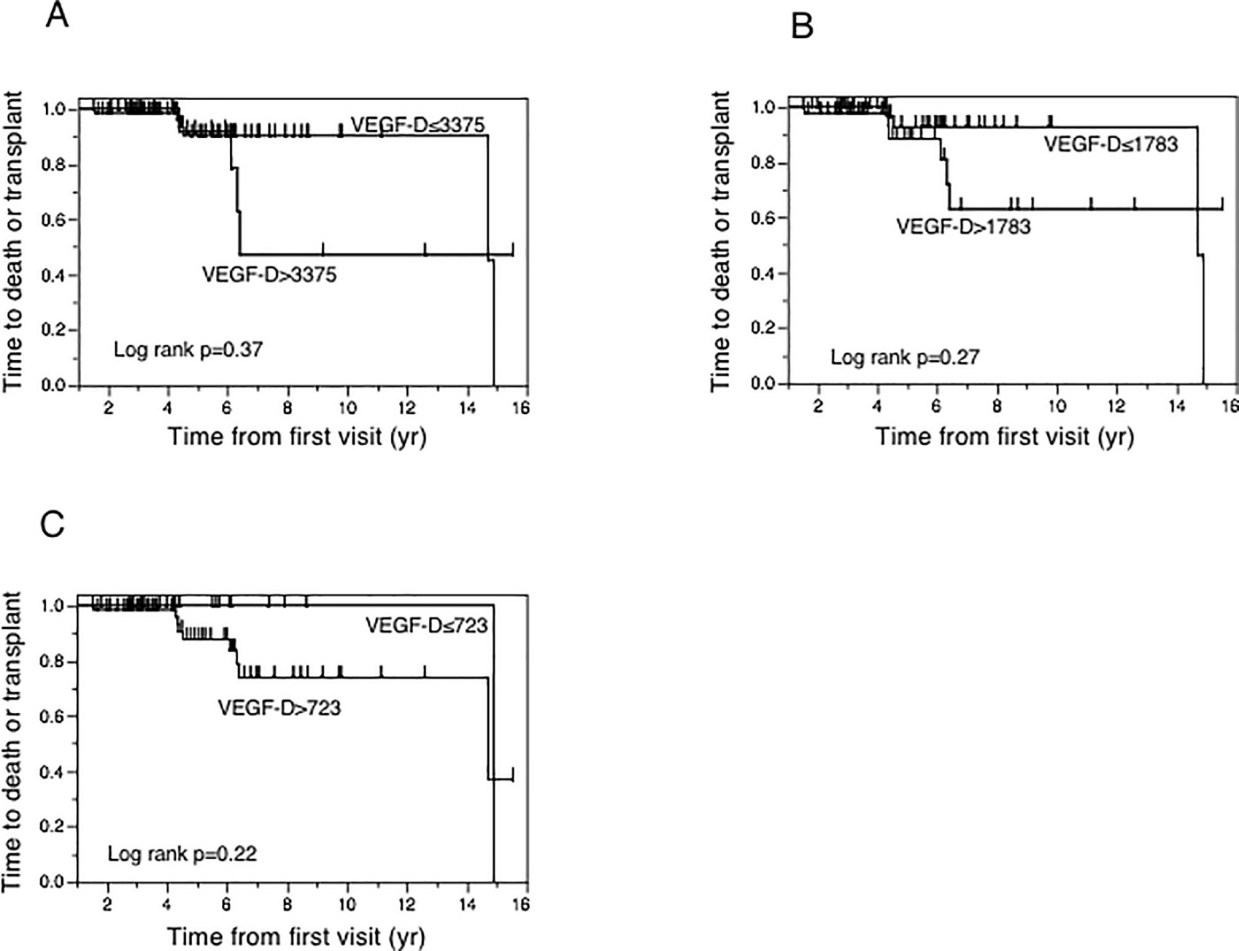
Survival rate and the baseline serum VEGF-D levels (interquartile range) in LAM. There was no significant association between baseline serum VEGF-D level and prognosis (death and lung transplantation).

The median follow-up period enrolled subjects was 3.6 years (range, 1.6–4.8 years; Table 1). During the study period, eight patients died, six underwent lung transplantation, and one died following lung transplantation. We divided the LAM cohort into quintiles and compared the outcomes of patients with baseline VEGF-D levels in the highest quintile (> 3375 pg/ml, n = 22/87, p = 0.37, Fig 4A), middle quintile (1783 pg/ml, p = 0.27, Fig 4B), lowest quintile (< 723 pg/ml, n = 21/87, p = 0.22, Fig 4C). There was no significant association between baseline serum VEGF-D level and prognosis (death or lung transplantation) in this analysis.

### Longitudinal assessment of serum VEGF-D levels over time

**Fig 5:**
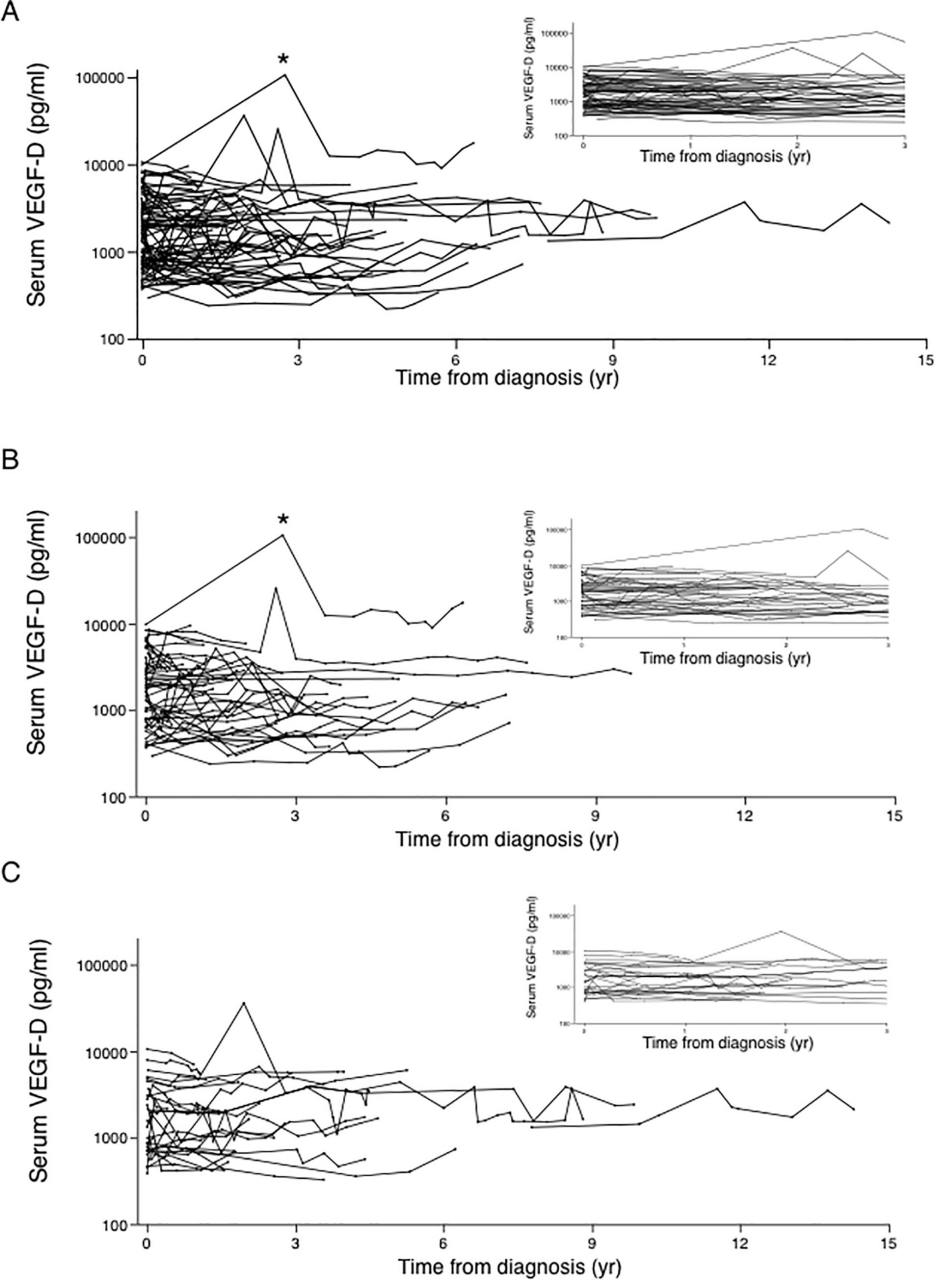
Longitudinal serum VEGF-D levels in LAM patients not receiving mTOR inhibitor therapy. Each line represents an individual patient. Panel (A) shows data from years 0 to 15 (inset is a close-up of years 0 to 3). Data for premenopausal (panel B) and postmenopausal (panel C) patients are also shown. To be included in the postmenopausal group, subjects must have undergone natural menopause or menopause caused by gonadotropin-releasing hormone analogues prior to the first visit to our institution. * denotes timing of acute AML hemorrhage.

Fig 5 shows serum VEGF-D levels only in the periods when 98 LAM patients out of 108 LAM patients were not taking mTOR inhibitors. The serum VEGF-D levels of these untreated S-LAM (n = 88) and TSC-LAM (n = 10) patients were remarkably stable throughout the study (Fig 5A), and whether the patients were pre- (Fig 5B) or post-menopausal (Fig 5C). Interestingly, one patient with TSC-LAM who experienced an acute AML hemorrhage exhibited a dramatic but transient increase in her serum VEGF-D level (Fig 5A and 5B).

### Effect of mTOR inhibitors on serum VEGF-D levels

**Fig 6:**
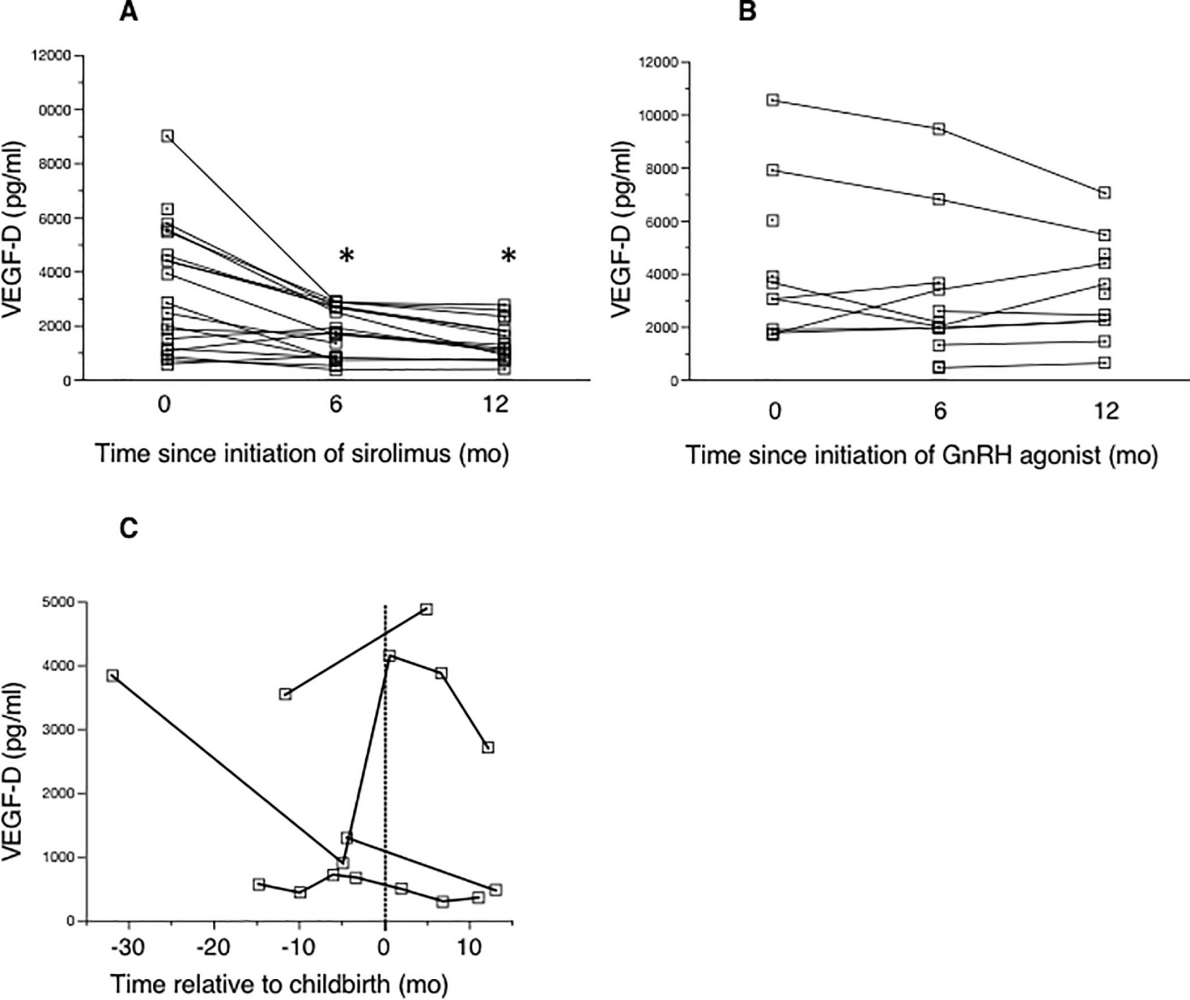
Effect of treatment on serum VEGF-D levels. (A) Serum VEGF-D levels significantly decreased after 6 (p < 0.05) and 12 months (p < 0.05) of sirolimus treatment compared with baseline levels (n = 12). (B) Serum VEGF-D levels before treatment with gonadotropin-releasing hormone (GnRH; n = 10) and after 6 (n = 12) and 12 (n = 11) months of treatment. (C) Changes in serum VEGF-D levels before and after pregnancy (n = 4).

In our cohort, 35 patients with LAM received sirolimus therapy during the study period, at dosages of 1–4 mg daily. Among them, 15 received sirolimus for more than 12 months. Serum VEGF-D levels significantly decreased in the first six months of treatment (Fig 6A) and continued to decline gradually as drug exposure continued. Because this study was retrospective, serum samples were not obtained at defined intervals; the number of patients with serum levels measured at the uniform time points of 0, 6, and 12 months after treatment began were limited to 19, 18, and 15, respectively (Fig 6A). In this subset, serum VEGF-D levels decreased significantly after 6 and 12 months of sirolimus treatment compared to baseline levels (p < 0.05 each). However, there was no significant difference between serum VEGF-D levels at 6 and 12 months following the introduction of sirolimus treatment, suggesting VEGF-D levels reach their minimum within the first six months of therapy.

### Effect of GnRH agonists and analogues on serum VEGF-D levels

The effect of GnRH agonists and analogues on serum VEGF-D levels was serially evaluated in 12 LAM patients. Fig 6B suggests that, in most cases, serum VEGF-D levels were stable before and during GnRH treatment, but because of the small sample size and non-uniform sample collection intervals, statistical analysis was not attempted. For a few patients with markedly elevated serum levels of VEGF-D, these levels appeared to decrease with GnRH treatment. In this study, pulmonary functions were stable before and after GnRH treatment.

### Effect of pregnancy on serum VEGF-D levels

The effect of pregnancy on serum VEGF-D levels was evaluated in four S-LAM patients. Serum VEGF-D levels remained largely unchanged during pregnancy in three of these patients. However, in one patient, the serum VEGF-D level increased dramatically around the time of childbirth, followed by a gradual decrease (Fig 6C).

## Discussion

In this study, we assessed the utility of serum VEGF-D as a diagnostic and therapeutic biomarker in Japanese patients. We found baseline serum VEGF-D levels effectively distinguished LAM from healthy volunteers and patients with other lymphatic and cystic lung diseases, with a sensitivity and specificity of 0.83 and 0.97, respectively, at a cut-off level of 645 pg/ml. At 800 pg/ml serum VEGF-D was slightly less sensitive (0.70) but was diagnostically specific (0.90) [16]. Therefore, as in the U.S., serum VEGF-D levels of 800 pg/ml can establish the diagnosis of LAM in Japanese women with typical cystic changes on HRCT with nearly 100% specificity [6].

In the longitudinal analysis, serum VEGF-D levels remained stable in patients not receiving sirolimus therapy during the study period. Sirolimus therapy significantly decreased serum VEGF-D levels, while GnRH therapy did not produce any consistent effects. Similarly, neither pregnancy nor menopause appeared to influence VEGF-D levels in a consistent manner.

### Serum VEGF-D levels in healthy volunteers

Although serum VEGF-D is a sensitive and specific biomarker for the diagnosis of LAM [5, 11, 17], the reported normative ranges for serum VEGF-D levels vary. The reported ranges have included mean serum VEGF-D levels of 296 pg/ml (262.6–333.5 IQR) in Japan [17], 657 ± 43 pg/ml (mean ± SE) at the National Institutes of Health, Bethesda, Maryland, USA [11], 309 pg/ml (211–433 IQR) in Cincinnati, Ohio, USA [6], 671.1 ± 501.8 pg/ml (mean ± SE) in Oman [21], 332 pg/ml (median) in the U.K. [22], and 405.5 pg/ml (245.3–527.8 IQR) in China [10]. The manufacturer of the serum VEGF-D test kit that we used (R&D Systems Inc.) lists a mean serum VEGF-D level of 297 pg/ml (range, 153–642 pg/ml). This distribution of reported means/medians is bimodal, with four laboratories and the manufacturer reporting values ranging from approximately 300–400 pg/ml and two other laboratories reporting means of approximately 650 pg/ml. This suggests that differences in sample handling, standard preparation, or other aspects of assay conduct could be responsible for the variability. If measuring serum VEGF-D levels is to become standard practice for diagnosing LAM, we must ascertain the basis of these differences. For this reason, as a first step, we compared serum VEGF-D level results from Osaka MILES samples tested using the same kit (R&D Systems Inc.) at both the TTDSL CAP/CLIA laboratory in Cincinnati and in our laboratory in Osaka. There was excellent agreement between measurements from the two laboratories (Fig 1), suggesting that international test performance is similar and that serum VEGF-D levels are similar in white and Asian individuals. The results of our analysis also lend support to a cut-off value of 800 pg/ml as a diagnostically specific threshold for LAM in women with typical cystic changes. This is the value originally proposed by Young et al. [5, 6] and validated by investigators in China [10], Poland [23], and the U.K. [22]. Although test performance of lower VEGF-D level cut-offs, such as 600–640 pg/ml, is strong, the higher threshold of 800 pg/ml is favored to minimize false positives, which could lead to inappropriate treatment with mTOR inhibitors [15, 16]. In addition, inter-assay variability of VEGF-D is higher at lower concentrations which further supports assigning the threshold for clinical significance at 800 pg/dl. These data support the international implementation of the recent ATS/JRS LAM Clinical Practice Guidelines recommendation for using VEGF-D when diagnosing LAM [15, 16] and as an eligibility criterion in clinical trials, including international multicenter trials [8, 19].

### Association of baseline serum VEGF-D levels with clinical characteristics and outcomes

In our study, serum VEGF-D levels were elevated in patients with lymphangioleiomyomas as previously reported [8, 11], but not in those with other lymphatic manifestations, such as chylous effusions (Table 2). The published literature contains conflicting data regarding whether VEGF-D levels are related to the presence of AML [6, 11, 13]. While our study found no relationship between elevated VEGF-D levels and the presence AML, there was a trend toward higher serum VEGF-D levels in TSC-LAM compared with S-LAM patients, which is consistent with previously reported findings [6]. Baseline serum VEGF-D levels were negatively correlated with baseline DL_co_ and yearly percent decrease in DLco, but not with other pulmonary function parameters (Table 3), consistent with previous reports [11]. There was no significant association between baseline serum VEGF-D level and prognosis. To clarify the potential prognostic significance of serum VEGF-D as a prognostic biomarker, further study in a larger trial is required.

### Natural progression of serum VEGF-D levels in our Japanese cohort

We found that serum VEGF-D levels did not increase in LAM patients as the disease progressed, as typically occurs with conventional tumor markers [24, 25]. VEGF-D levels were in fact remarkably stable over time in both pre- and post-menopausal patients. Given that VEGF-D is thought to be produced by LAM cells, the increase in tumor activity over time might have been expected to result in higher growth factor levels. It is interesting that serum VEGF-D levels spiked during an AML hemorrhage in one patient (Fig 5A), suggesting that the may be a source of VEGF-D and that the growth factor may gain access to circulation during vascular events associated with permeability, ischemia, or necrosis.

### Effect of estrogen and mTOR inhibitors on serum VEGF-D levels

The MILES trial revealed that sirolimus stabilized pulmonary function and decreased serum VEGF-D levels in LAM patients [8]. Our study lends support to these findings, showing a decrease in serum VEGF-D levels upon treatment with sirolimus (Fig 6A). Serum VEGF-D levels did not change in a consistent manner during GnRH agonist therapy, pregnancy, or menopause, suggesting that estrogen does not modulate the levels of this growth factor. It is important to note that the ATS/JRS Guidelines currently recommend not using hormonal therapy for treating LAM [15, 16].

### Limitations of the study

There were several limitations in this retrospective, single-center cohort study (Japanese data), including the small number of patients with other lung diseases, the inter-assay variability, and the small number of lung transplantation and death events.

## Conclusion

In summary, we confirmed that serum VEGF-D is a useful diagnostic and prognostic biomarker in the Japanese patient population. We also found that serum VEGF-D levels tend to be remarkably consistent over time, and that neither menopausal status nor hormonal fluxes during pregnancy significantly modulate VEGF-D levels, although further studies are needed to confirm these relationships testing. We concluded that serum VEGF-D levels are a globally useful diagnostic and therapeutic biomarker for LAM. Satisfactory precision and international inter-laboratory agreement of the clinical assay support the application of ATS/JRS LAM Guidelines in the Japanese population.

## Supporting information

S1 Fig Serum level of VEGF-D in 108 patients with LAM (sporadic,92; TSC,16) compared to 35 other lung diseases (OLDs) and 76 healthy controls. Dotted line shows 800 pg/ml. Control (F), healthy female controls; Control (M), healthy male controls; S-LAM, Sporadic-LAM; N.S, not significant.
(TIFF)
