# Supplementary figures and images for: Serum vascular endothelial growth factor-D as a diagnostic and therapeutic biomarker for lymphangioleiomyomatosis
Source: PLoS One. 2019 Feb 28;14(2):e0212776. doi: 10.1371/journal.pone.0212776 (PMC6395035; doi:10.1371/journal.pone.0212776)

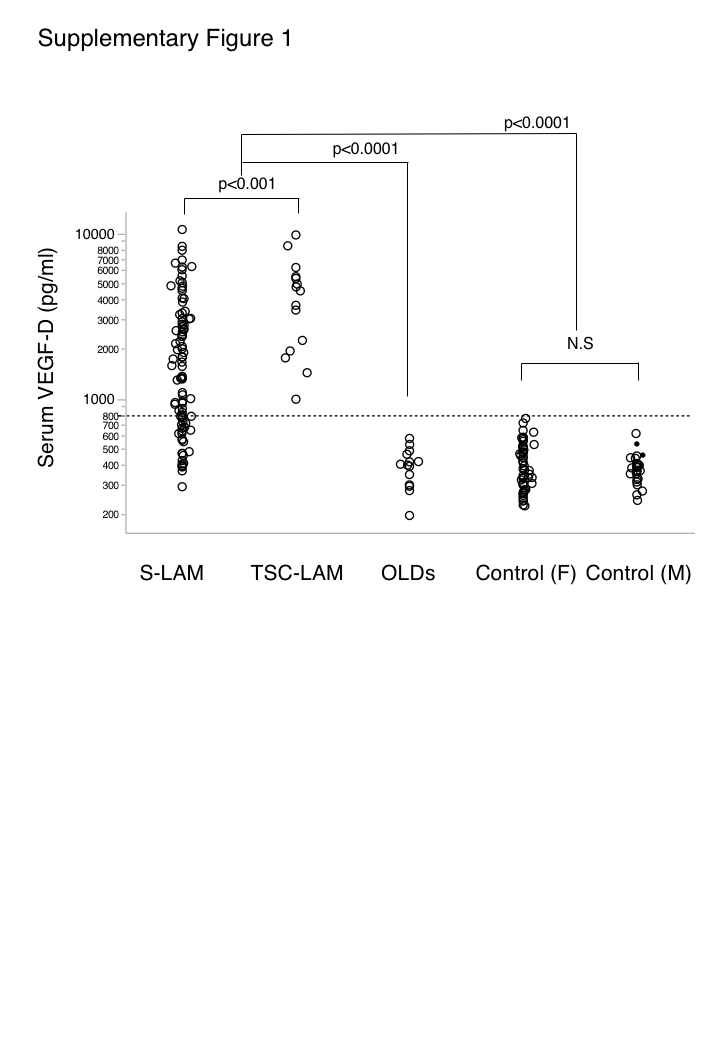

Supplement: S1 Fig — Dotted line shows 800 pg/ml. Control (F), healthy female controls; Control (M), healthy male controls; S-LAM, Sporadic-LAM; N.S, not significant. (TIFF) [file pone.0212776.s001.tiff]
